# Metal toxicity differently affects the *Iris pseudacorus*-arbuscular mycorrhiza fungi symbiosis in terrestrial and semi-aquatic habitats

**DOI:** 10.1007/s11356-015-5706-x

**Published:** 2015-11-20

**Authors:** K. Wężowicz, K. Turnau, T. Anielska, I. Zhebrak, K. Gołuszka, J. Błaszkowski, P. Rozpądek

**Affiliations:** Institute of Environmental Sciences, Jagiellonian University, Gronostajowa 7, Kraków, 30-387 Poland; Institute of Plant Physiology, Polish Academy of Sciences, Niezapominajek 21, Kraków, 30-239 Poland; Malopolska Centre of Biotechnology, Jagiellonian University, Gronostajowa 7A, Kraków, 30-387 Poland; Department of Plant Protection, West Pomeranian University of Technology, Słowackiego 17, Szczecin, 71-434 Poland; Department of Botany, Yanka Kupala State University, Grodno, Belarus

**Keywords:** *Iris pseudacorus*, Arbuscular mycorrhizal fungi (AMF), Toxic metals, Constructed wetlands, Phytoremediation, OJIP

## Abstract

Phytoremediation offers an environmental friendly alternative to conventional cleanup techniques. In this study, mycorrhizal fungi isolated from the roots of *Mentha longifolia* grown in the basin of the Centuria River (S Poland) were used. *Iris pseudacorus* was grown in substratum from an industrial waste, enriched in Pb, Fe, Zn, and Cd in a terrestrial and water-logged habitat. Plant yield and photosynthetic performance was the highest in the aquatic environment; however, the presence of toxic metals (TM) negatively affected photosystem II (PSII) photochemistry as shown by the JIP test. Fungi colonization and Cd accumulation within plant tissues was decreased. In the terrestrial habitat, neither arbuscular mycorrhizal fungi (AMF) nor metal toxicity affected plant growth, although metal uptake, Cd in particular, as well as photosynthesis were affected. Inoculated plants accumulated significantly more Cd, and photosynthesis was downregulated. The results presented in this study clearly indicate that the *I. pseudacorus*-AMF symbiosis adapts itself to the presence of toxic metals in the environment, optimizing resource supply, energy fluxes, and possibly stress tolerance mechanisms. Plant/AMF consortia grown in terrestrial and water-logged habitats utilize different strategies to cope with metal toxicity. The use of AMF in improving the phytoremediation potential of *I. pseudacorus* needs, however, further research.

## Introduction

Constructed wetlands offer a cost-effective, environmental friendly alternative to conventional cleanup techniques to treat domestic and industrial wastewater (Kivaisi 2001). The selection of plants for such a system can provide not only water quality improvement but also economic benefits through production of “bio-gas,” animal forage, compost, and the production of fiber (Lakshman 1987). The introduction of appropriately selected plants into such sites may not only have practical significance in waste treatment but also can allow the improvement of the aesthetic appearance of the landscape.

Industrial wastes, which are an integral part of the landscape of South Poland and many other European countries, are often inhabited by plant species tolerant to a wide amplitude in terms of water relations. So far, the role of arbuscular mycorrhiza fungi (AMF) in improving plant fitness and remediation techniques has not been tested in aquatic or water-logged habitats, even though their utility in re-cultivating terrestrial environments has been shown previously (Turnau and Haselwandter 2002; Turnau et al. 2005; Turnau et al. 2006; Turnau et al. 2012). In such cases, mycorrhiza has been shown to improve plant growth, rooting, to attenuate drought and toxic metal stress, improve stability of the substratum, and decrease wind and water erosion.

So far, plants considered for re-cultivation in constructed wetlands were indicated as non-mycorrhizal due to the characteristic of the substratum. When the water level rises—growth of such fungi was believed to be strongly limited or inhibited, but propagules were shown to persist (Mejstrik 1972, Dolinar and Gabarscik 2010). Recently, this opinion has been questioned. Miller and Sharitz (2000) and Wang et al. (2015) found that flooding may not limit the development of AMF. Several reports concerning mycorrhizal fungi development in plants from aquatic and semi-aquatic habitats exists (e.g., Nielson et al. 2004; Wirsel et al. 2004; Bohrer et al. 2004; Wang et al. 2011), although, the biology of these fungi is largely unknown. Out of the few known facts, AMF rather decrease plant diversity under water-logged conditions (Wolfe et al. 2006). At the same time, flooding decrease AMF diversity (Wang et al. 2011).

In recent years, we obtained AMF strains from *Mentha longifolia* roots from the riverbed of the Centuria River, close to Olkusz (Poland). The roots of this plant were strongly colonized by AMF and the maintenance of the inoculum was possible over the last 5 years in pot cultures in an aquatic environment (data unpublished).

*Iris pseudacorus* is well known for its ability to inhabit terrestrial and temporarily (wetlands) flooded sites. It was previously shown valuable as a phytoremediating plant in wastewater or wetland treatment, for its ability to remove organic matter (Ansola et al. 1995), Pb (Han et al. 2008), Cd (Han et al. 2007), and Cr (Wolfe et al. 2006; Caldelas et. al. 2012). According to Zhang et al. 2007, *I. pseudoacorus* removes up to 96 % of toxic metals from sewage. However, according to Zhou et al. (2010) its growth and vitality is severely impaired by Pb and Cd.

The aim of this study was (i) to explore the potential of AMF in re-cultivating temporarily flooded post-industrial wastes (enriched in toxic metals); (ii) to estimate the impact of AMF on toxic metal uptake depending on the water conditions; (iii) to evaluate the vitality of plants depending on presence/absence of AMF and heavy metals; (iv) to gain more insight on the biology of AMF in aquatic and water-logged habitats.

## Methods

### Plant and fungal material

Rhizomes (8–9 cm long) of *I. pseudacorus* L. were bought in the internet shop Oczko wodne (www.sklep.oczkowodne.net). The inoculum was obtained from roots of *M. longifolia* collected from the Centruria source water near Olkusz (Poland). Trap cultures were set up in 1000 ml pots on sterile substratum containing sand and expanded clay (2:1, *v*/*v*), maintained in standing water. *Plantago lanceolata* was used as the host plant. The following AMF were identified (according to morphological traits) from trap cultures: *Diversispora epigaea* (Daniels and Trappe 1979; Schüßler et al. 2011), *Glomus aureum* (Oehl et al. 2003), *Rhizophagus irregularis* (Błaszkowski et al. 2004; Schüßler et al. 2011), and *Rhizophagus clarus* (Nicolson and Schenck 1979; Schüßler et al. 2011). After 6 months of cultivation, shoots were harvested while root samples were subjected to staining according to the Trouvelot method (Trouvelot et al. 1986). Over 80 % of *P. lanceolata* root was colonized by AMF; no other root endophytes were identified in the material.

### Experiment design

Rhizomes of *I. pseudacorus* were planted in pots (2000 ml) in sterile substratum, a single plant per pot, for the toxic metal treatment, substratum from the Trzebionka post-flotation waste (Table 1). Total metal content of the substratum was analyzed with AAS (Varian 220FS). The pH of the substratum was measured at 7.4 in H_2_O.The substratum exhibited extractable quantities shown to limit plant growth (Szarek-Łukaszewska 2009). For control, sand and volcanic clay were used. Plants were inoculated with equal volumes (50 g) of dried inoculum. Additionally, autoclaved fungal inoculum (AMF laboratory strains) was added to the control. Plants were cultivated in pots kept in larger containers filled with water into half volume. For “terrestrial” conditions, the moisture of the substratum was maintained at 0.14 and strictly controlled twice a week using moisture meter. To evaluate the role of AMF in improving plant vitality under the presence of increased concentrations of toxic metals in the substratum, the following treatments were included: *W*−*M*−*I*−, *W*−*M*+*I*−, *W*−*M*+*I*+, *W*−*M*−*I*+, *W*+*M*+*I*+, *W*+*M*−*I*+, *W*+*M*−*I*−, *W*+*M*+*I*−, where: W− terrestrial plants, *W*+ water-logged plants, *M*− sand, *M*+ toxic metal-enriched substratum, *I*− non-inoculated plants, and *I*+ plants inoculated with AMF. The experiment was repeated three times. In each experiment, five to nine replicates were established (depending on the treatment). The full data set was collected from the last experiment. Plants were grown under greenhouse conditions at 20 °C average air temperature, at light intensity *ca.* 200 μmol × m^−2^ × s^−1^ and a 12-h photoperiod for 3 weeks and subsequently moved outside the building into the garden (field conditions). Plants were harvested after a 5-month growing period.

**Table 1:** Total (in HClO_4_) and extractable (in 1 M NH_4_NO_3_ and 0.1 M Ca(NO_3_)_2_) element content in the substratum; data in micrograms per gram (Orłowska et al. 2005)

Element	Total extracted in HClO_4_	Extracted in NH_4_NO_3_	Extracted in Ca(NO_3_)_2_
Cd	225	1.9	1.2
Pb	2790	0.9	3.03
Zn	15,524	37	2.4
P	900		
N	900		
C	60,500		

### Mycorrhiza evaluation

The roots were washed with tap water and stained for estimation of mycorrhizal parameters according to the modified method by Phillips and Hayman (1970). Briefly, after washing in tap water, the roots were softened in 10 % KOH for 24 h, washed in water, acidified in 5 % lactic acid for 1 h at room temperature, and stained in 0.01 % aniline blue in pure lactic acid for 24 h. After staining, the roots were stored in pure lactic acid. The roots were cut into 1 cm pieces, mounted on slides in glycerol, and analyzed. Relative mycorrhizal root length (*M*), relative arbuscular richness (*A*) were assessed according to Trouvelot et al. (1986). Prior to vitality and metal content analysis, all plants were evaluated for the presence of mycorrhiza. Control plants (non-inoculated with laboratory inoculum) that showed presence of AMF were not subject to further analysis. For each treatment, at least 300 root pieces were evaluated.

### Toxic metal concentration determination

*I. pseudacorus* roots and shoots were washed with redistilled water, dried at 85 °C and weighed with an accuracy of 0.01 g. The dried and milled plant tissue was subsequently mineralized (digested) in a 4:1 mixture of ultra-pure concentrated HNO_3_ and HClO_4_ (MERCK) (Pinta 1977; Grodzińska 1978). Fe, Zn, Cd, and Pb content in plant roots and shoots were analyzed with FAAS (Varian 20BQ), with the following limits of detection (LOD): Fe 0.171 mg/dm^3^, Zn 0.011 mg/dm^3^, Cd 0.024 μg/dm^3^, Pb 0.530 μg/dm^3^. For reference, SRM 1575a—Trace Elements in Pine Needles https://www-s.nist.gov/srmors/view_detail.cfm?srm=1575a) were used.

### Evaluation of plant vitality

To evaluate plant vitality, OJIP chl *a* fluorescence transients were measured with the HandyPEA-fluorimeter (Hansatech Instruments, King’s Lynn Norfolk, PE 30 4NE, UK). This method is broadly used to study plant performance under various stress conditions and as a tool to monitor plant health (Table 3). The transients were induced by red light (peak at 650 nm) of 3000 μmol photons m^−2^ s^−1^ provided by an array of three light-emitting diodes and recorded according to manufacturer instructions. The obtained data was analyzed with the BIOLYZER v.5.0 Professional (Fluoromatics Software, Geneva, Switzerland). The measurements were carried out on the second youngest leaf after dark adaptation with clips provided by the manufacturer for 30 min prior to measurements.

### Statistical analysis

All data was evaluated for homogeneity of variance normal distribution prior to ANOVA. If the analyzed data did not meet the conditions, the log transformation was carried out. The Duncan post hoc test was carried out (*p* < 0.05) using STATISTICA v. 7.1 (Statsoft).

## Results

### AMF colonization

**Fig. 1:**
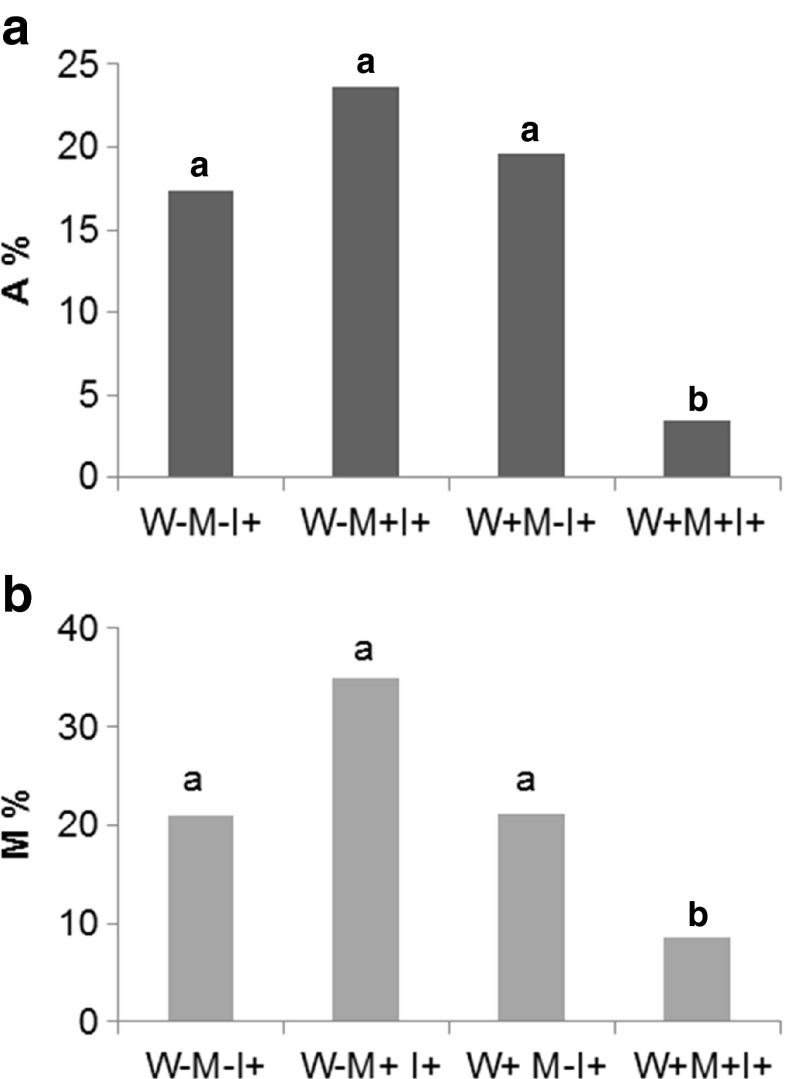
**a** Relative arbuscular richness (*A*%) of aniline blue-stained *I. pseudacorus* roots. *W−* terrestrial plants, *W+* water-logged plants, *M−* sand, *M+* toxic metal-enriched substratum, *I−* non-inoculated plants, *I+* plants inoculated with AMF. Different letters above bars indicate statistically significant differences (*P* < 0.05). *N* = 9. **b** Relative mycorrhizal root length (*M*%) of aniline blue-stained *I. pseudacorus* roots. *W−* terrestrial plants, *W+* water-logged plants, *M−* sand, *M+* toxic metal enriched substratum, *I−* non-inoculated plants, *I+* plants inoculated with AMF. Different letters above bars indicate statistically significant differences (*P* < 0.05). *N* = 9

The analyzed non-inoculated plants did not show any mycorrhizal structures. Inoculated plants exhibited a typical *Arum* type mycorrhiza with well-developed arbuscules. The presence of toxic metals in the substratum did not change *A*% or *M*% in the terrestrial habitat. Water-logging resulted in a significant decrease in *A*% and *M*% in the presence of toxic metals. Both indices where similar for plants grown in different habitats (Fig. 1a, b). No additional fungi (such as DSE) were found in the studied material.

### Plant growth

**Fig. 2:**
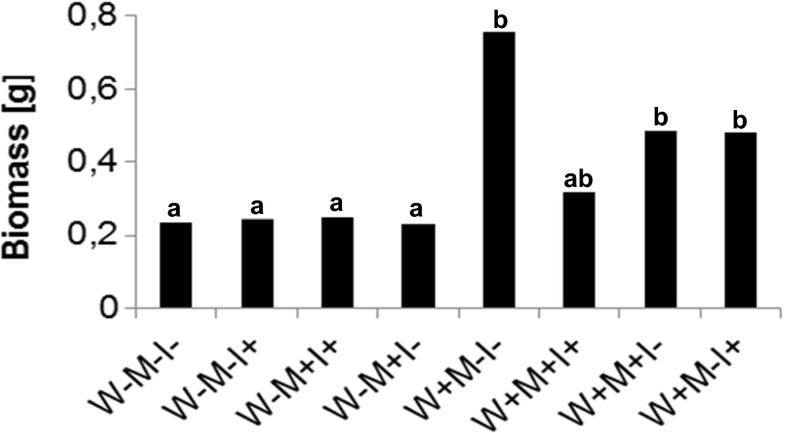
Biomass of *I. pseudacorus* cultivated under different water regimens. *W−* terrestrial plants, *W+* water-logged plants, *M−* sand, *M+* toxic metal enriched substratum, *I−* non-inoculated plants, *I+* plants inoculated with AMF. Different letters above bars indicate statistically significant differences (*P* < 0.05). *N* = 9

Water-logged *I. pseudacorus* yielded significantly more biomass (Fig. 2). The weight of *M*−*W*+*I*− plants averaged 757.25 mg compared with 235.875 *M*−*W*−*I*−. No significant difference was shown in plants grown in the terrestrial habitat (app. 236 ± 8 mg). In water-logged conditions, both AMF and TM decreased the mean biomass of *I. pseudacorus*, although the differences were statistically insignificant. The results were consistent in all three experiments.

### Metal concentration in mycorrhizal and nomycorrhizal *I. pseudacorus*

Neither water-logged conditions nor AMF had any effect on the concentration of Fe and Pb in *I. pseudacorus*. The concentration of accumulated Zn increased by over 230 % in water-logged conditions. Inoculation did not significantly affect it. Terrestrial plants accumulated less Cd compared with their aquatic counterparts; however, AMF inoculation resulted in significant alterations in Cd uptake. In *I. pseudacorus* grown in the “land,” colonization significantly (by 64 %) increased Cd concentration in plant tissues. On the contrary, after water-logging, the concentration of Cd decreased after inoculation by 19 % (Table 2).

**Table 2:** Accumulation of Fe, Pb, Zn, and Cd in *I. pseudacorus*

Treatments	Pb	Cd	Fe	Zn
*W*−*M*−*I*−	0.077 a	0.080 a	46.319 a	33.228 a
*W*−*M*−*I*+	0.136 a	0.302 a	70.342 a	38.389 a
*W*−*M*+*I*+	0.121 a	2.769 c	87.223 a	209.635 b
*W*−*M*+*I*−	0.116 a	1.795 b	75.729 a	199.723 b
*W*+*M*−*I*−	0.100 a	0.092 a	62.122 a	45.443 a
*W*+*M*−*I*+	0.078 a	0.088 a	79.105 a	52.002 a
*W*+*M*+*I*+	0.113 a	2.844 c	75.632 a	464.007 c
*W*+*M*+*I*−	0.174 a	3.472 d	77.484 a	470.526 c

Data in μg/g. Different letters next to metal content indicate statistically significant differences (*P* < 0.05)

*W−* terrestrial plants, *W+* water-logged plants, *M−* sand, *M+* toxic metal-enriched substratum, *I−* non-inoculated plants, *I+* plants inoculated with AMF

### Photosynthesis efficiency

**Fig. 3:**
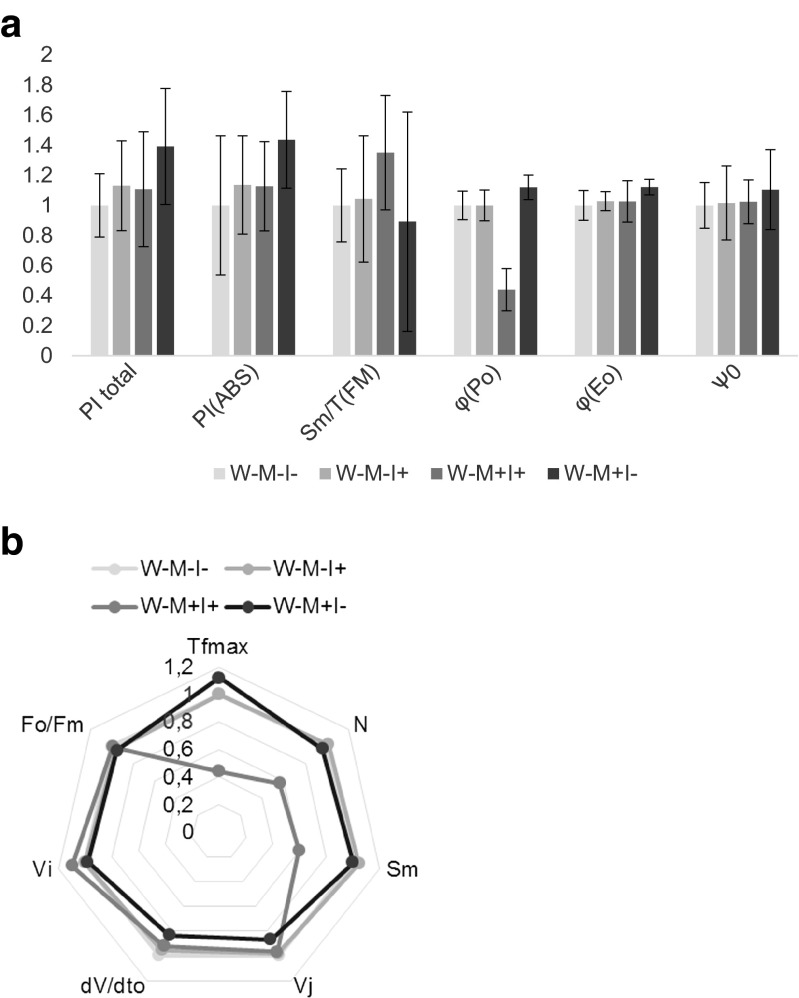
**a** PI_total_, PI_ABS_, and *S*_m_/*T*_FM_ φ_P0_ φ_E0_ ψ_0_ of *I. pseudacorus* from the terrestrial habitat. *W−* terrestrial, *M−* sand, *M+* toxic metal enriched substratum, *I−* non-inoculated plants, *I+* plants inoculated with AMF. Definitions of the parameters are listed in Table 3. *N* = 5. **b** “Spider plot” of selected fluorescence parameters describing energy fluxes in photosystem II. *W−* terrestrial plants, *M−* sand, *M+* toxic metal-enriched substratum, *I−* non-inoculated plants, *I+* plants inoculated with AMF. Definitions of the parameters are listed in Table 3. *N* = 5

**Fig. 4:**
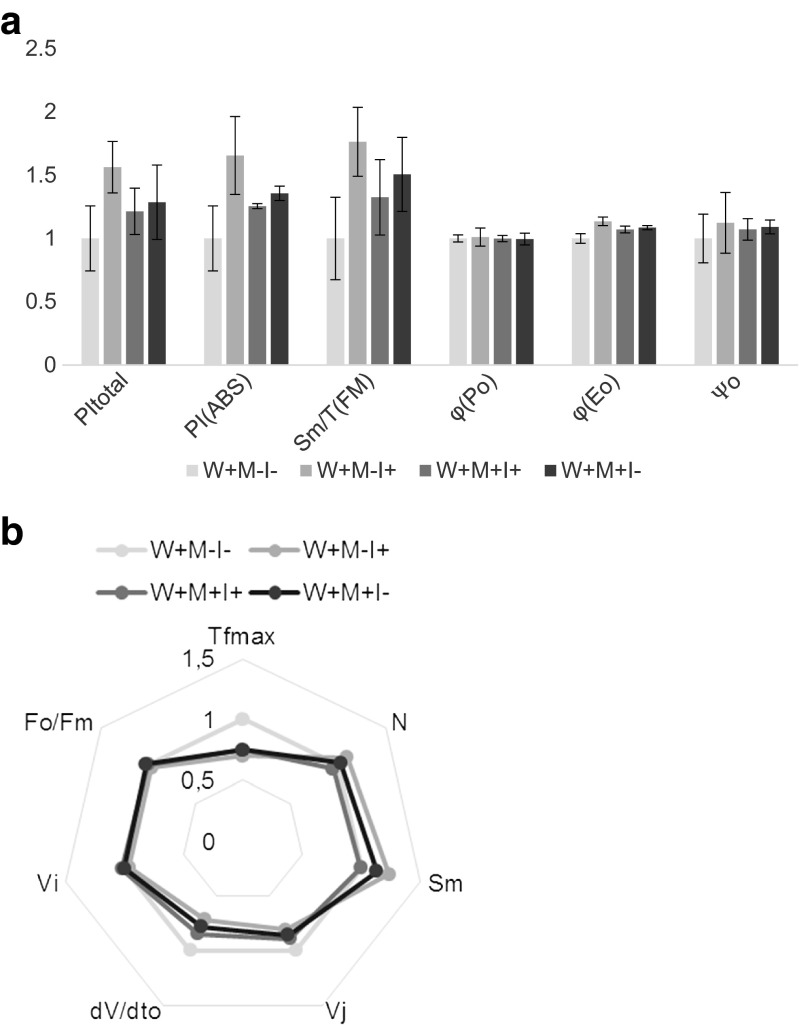
**a** PI_total_, PI_ABS_, and *S*_m_/*T*_FM_ φ_P0_ φ_E0_ ψ_0_ of *I. pseudacorus* from the water-logged habitat. *W+* water-logged plants, *M−* sand, *M+* toxic metal enriched substratum, *I−* non-inoculated plants, *I+* plants inoculated with AMF. Definitions of the parameters are listed in Table 3. *N* = 5. **b** “Spider plot” of selected fluorescence parameters describing energy fluxes in photosystem II. *W+* water-logged plants, *M−* sand, *M+* toxic metal-enriched substratum, *I−* non-inoculated plants, *I+* plants inoculated with AMF. Definitions of the parameters are listed in Table 3. *N*=5

According to performance indexes PI_total_ and PI_ABS_ (Table 3) photosynthesis efficiency was improved in *I*+ plants by 55 and 65 %, respectively, at water-logged conditions. Surprisingly, the presence of TM improved the performance indexes by 28–36 % in *I*− and 19–25 % in *I*+ (Figs. 3a and 4a).

**Table 3:** Terms used in the analysis of the OJIP fluorescence transient

Symbol	Description
d*V*/d*t*_0_	Initial slope of relative fluorescence. Directly describes the trapping flux TR_0_/RC
S_m_	Energy needed to close all reaction centers
*N*	Turn-over number. Indicates how many times QA can be reduced in a time spam from 0 to *T*_FM_
*V*j	Relative fluorescence emission during the J phase (2 ms). Corresponds to the maximal rate of the accumulation of the fraction of closed reaction centers
*V*i	Variable fluorescence at I step
S_m_/*T*_FM_	The average redox state of QA in the time span from 0 to *T*_FM_ and, concomitantly, the average fraction of open reaction centers during the time needed to complete their closure
*T*_FM_	Time to reach the maximal fluorescence
Ѱ_0_ (ET_0_/TR_0_)	Quantum yield of electron transport
ϕP_0_ (TR_0_/ABS)	the maximal yield of primary photochemistry. When calculated from extreme values (F_0_ and Fm) Fv/Fm
ϕE_0_ (ET_0_/ABS)	The probability that an absorbed photon will move an electron into the electron transport chain
PI total	Multiparametric expression illustrating photosynthetic activity of a RC complex. Accounts for absorption of light energy (ABS), trapping of excitation energy (TR), conversion of excitation energy to electron transport (ET) and reduction of end acceptors (RE)
PI _ABS_	The performance of the photosynthesis apparatus expressed in relation to absorption. Acounts for the density of reaction centers, the quantum yield of primary photochemistry and the ability to transfer electrons from PSII to PSI

In *W*− plants, mycorrhiza had no effect on the performance indexes, but the presence of TM in the substratum (37–44 %) improved it (Fig. 3a). Quantum efficiencies and flux ratios (Ѱ_0_, ϕP_0_, ϕE_0_) were not affected by either AMF or TM under both water regimens (Figs. 3a and 4a). Under water-logged conditions, *S*_m_/*T*_FM_ was improved by both AMF (76 %) and the presence of TM (50 %), but it was lower, however, in *M*+*I*+ plants compared with *M*+*I*− by 18 % (Fig. 4b). *S*_m_/*T*_FM_ was not affected in plants from the terrestrial habitat (Fig. 3b).

In water-logged conditions *T*_FM_ decreased (Fig. 4b) upon inoculation (31 %) and TM (25 % for both *I*+ and *I*+). In terrestrial conditions, the presence of TM in the substratum had no effect on *T*_FM_, independently of the presence of AMF, but inoculation significantly decreased *T*_FM_ in plants grown in control substratum (Fig. 3b).

The fluorescence parameters *N* and *S*_m_ (Figs. 3b and 4b). A 46 and 41 % decrease was respectively reported. However, in water-logged conditions, no differences in *N* and a 23 % increase in *S*_m_ was shown (Fig. 4b). The initial slope of relative fluorescence (d*V*/d*t*_0_) was decreased in *M*+ plants grown in the aquatic habitat (28 %). In TM-treated plants, a similar response was reported, but this decrease was less prominent (Fig. 4b). In plants grown in the terrestrial habitat, the presence of TM in the substratum resulted in a decrease in trapping flux. No differences in this parameter were shown for *I*+ plants independently of growth conditions. Variable fluorescence during phase J (*V*_j_) was lower in *I*+ plants in water-logged conditions (20 %); however, metal toxicity increased it. In *M*+ plants, it reached 86 and 89 % in *I*+ and *I*−, respectively (Fig. 4b). The results were consistent in all three experiments.

## Discussion

In the present study, we investigated the biology of the plant/AMF symbiosis in *I. pseudacorus* and its potential role in alleviating metal-induced stress in plants under different water regimens. AMF were able to survive in an aquatic habitat for several years, thus be potentially used in e.g., constructed wetlands to support plant vitality and the remediation process. Our data support the findings of Wang et al. (2015) who studied mycorrhizal colonization of *Phragmites australis* from a mangrove swamp in the temperate continental monsoon climate zone (China) and suggested that AMF were able to adapt to water-logging. In this study, we confirm that AMF such as the cosmopolitan *R. irregularis* have the ability to adapt to flooded habitats. Traits such as TM tolerance are strain specific and can be lost if the fungi are not maintained in proper conditions (Sudowa et al. 2007). This is of crucial importance in the context of phytoremediation. Weather the ability of *R. irregularis* and other AMF to adapt to aquatic habitats is specific for certain strains remains open. Nevertheless, attention should be paid to maintain appropriate conditions in laboratory cultures.

In this study, we have shown that water-logging benefits the growth of *I. pseudacorus*. Plants associate with symbiotic organisms in order to gain an advantage in suboptimal habitats. One of these is limited access to nutrients such as P, N, which triggers mechanisms favoring the establishment of symbiosis (Azcón-Aguilar and Bago 1994). In this model, the plant/AMF consortium grown at different irrigation behaved differently in response to the presence of TM in the substratum. In preferable conditions (water-logged), the presence of TM in the substratum clearly decreased *M*% and *A*%. This may be a result of adaptation to TM toxicity. The availability of water and nutrients is higher in water-logged conditions. In the beneficial symbiosis, the fungi withdraw significant amounts of assimilated carbon from the plant in return supplying it with water and necessary nutrients. In this model, both water and necessary metals were at high abundance. As shown in Fig. 1b, mycorrhization was decreased in the presence of TM; however, it was sufficient enough to inhibit Cd accumulation indicating a protective role of the fungi. Decreased colonization indicates plant control of hyphal growth, optimizing it to its current needs. It seems as though supporting the fungi with reduced carbon became less feasible in the presence of TM stress in the aquatic habitat. As shown in Fig. 2, the biomass of *I*+ plants tended to decrease as well as several chl *a* fluorescence parameters, suggesting that the available carbon was utilized in processes other than provisioning the fungi, probably defense mechanisms. This however, needs confirmation. The performance indexes indicate that in the absence of TM, the plant can afford to support its fungal partner; however, when challenged by stress, it optimizes the interaction by limiting fungal growth. What is interesting the *A*% was unchanged confirming previous findings concerning the adaptation of fungi to water-logged conditions.

Even though mycorrhiza did not affect plant growth in *W*− *I. pseudacorus*, *M*% tended to increase in the presence of TM, indicating that such conditions are favorable for the interaction. As mentioned previously, in result of the AMF-plant interaction, growth of the partners is improved. Nutrient, water supply, and defense mechanisms are upregulated (Koide 1991; Sharma et al. 1992; Newsham et al. 1994; Smith and Read 1997), thus we propose that in the presence of TM the plant stimulates the growth of the fungi within its tissues in order to maintain homeostasis under metal toxicity. Performance indexes of inoculated plants do not change (comparing *M*+*I*+ with *M*−*I*+) under TM stress. Non-inoculated plants exhibited a significant increase in photosynthetic performance, suggesting the presence of different routes of adapting to metal toxicity in AMF plants and non-inoculated. AMF plants depended rather on fungi-dependent protection mechanisms; non-inoculated plants upregulated their metabolism in order to withstand metal toxicity. Additionally, Cd uptake in the terrestrial habitat was improved with no negative impact on plant growth, confirming the beneficial role of AMF. This however requires further studies.

It was shown previously that only AMF strains adapted to polluted environments positively impact plants under these specific conditions (Orłowska et al. 2005). The AMF inoculum used in this study originated from a non-polluted site, thus we assume that the little to no effect on metal uptake, growth, etc., might be due to the lack of adaptation of the fungi to metal toxicity. This seems to be the major drawback of the present study. AMF-induced stress alleviation has been shown mainly in terrestrial habitats, such as post-industrial waste slopes or industrial tailings (Turnau et al. 2012; Orłowska et al. 2013; Sprocatti et al. 2014) but so far there were no investigations concerning AMF in plants from constructed wetlands, although, the demand for improvement of water quality makes this subject important. *I. pseudacorus* has been well studied for its ability to grow in presence of heavy metals and our studies confirm its TM tolerance (Zhou et al. 2010; Caldelas 2012). To assist plant vitality, we used a widely accepted method that has a great potential to monitor not only the adaptation of the plant to a particular environment but also to estimate the role of microbiota in plant survival and effectiveness of metal detoxification. In this study it allowed us to link energy fluxes in the photosystem II (PSII) with the dynamics of the plant/AMF symbiosis in terrestrial and aquatic habitats under the presence of TM.

Mycorrhiza did not increase plant biomass (growth tended to be decreased in water-logged conditions) nor metal uptake by plants, even though Cd metabolism was clearly altered after inoculation. These features might not be of utmost important. We often consider mycorrhiza as a biomass stimulator or nutrient uptake enhancer and overlook its role as a heath and stability enhancer. Several parameters of photosynthesis were observed to be improved in the presence of AMF showing increased potential for energy conservation and CO_2_ assimilation.

While studying heavy metal impact on plant performance, mycorrhiza was found to be beneficial for the plant (e.g., Rivera-Beccerill et al. 2002). This study shows that plants inhabiting the riverbed associate with AMF. The ability of plants to maintain the symbiosis in water-logged conditions and to regulate colonization and Cd uptake in response to stress indicates high plasticity of the interaction. AMF does not improve the ability of *I. pseudacorus* to accumulate and/or withstand metal toxicity in the water-logged habitat. It even seems to be a handicap in such conditions; however, the plant is able to optimize colonization and subsequently metal uptake for sufficient energy and resource management. In the terrestrial habitat, colonization and Cd uptake increased suggesting a beneficial role of the fungi (metal stress defense, water and nutrient supply). In both cases, this plasticity leaves a fingerprint on the photosynthesis apparatus/electron transport in PSII.

## Conclusions

*I. pseudacorus* was shown to be able to develop and sustain a long-term association with AMF under water-logged conditions. Metal toxicity however, resulted in a decrease in colonization rate and Cd uptake. Interestingly, mycorrhiza was shown to improve Cd accumulation in the terrestrial habitat, indicating that *I. pseudacorus* employs different strategies in dealing with metal toxicity, depending on environmental conditions. It seems as though AMF may not be necessarily beneficial in improving metal cleanup in constructed wetlands, even though it was found to be beneficial for the plant when grown in an aquatic habitat. A long-term comprehensive study of AMF-plant physiology and ecology and elucidating the role of AMF in polluted aquatic and semi-aquatic habitats in the context of plant and microorganism biodiversity is necessary to draw final conclusions. This study may be a good starting point in understanding the biology of plant-AMF consortia in these specific conditions. Additionally, mycorrhizal strains adapted to metal toxicity may be a better fit for phytoremediation.
